# Case report: Subacute combined degeneration of the spinal cord due to nitrous oxide abuse

**DOI:** 10.3389/fneur.2023.1099077

**Published:** 2023-01-26

**Authors:** Huijuan Wu, Huiqing Huang, Liaoyang Xu, Niu Ji, Xinyu Zhou, Kang Xie

**Affiliations:** ^1^Department of Neurology, Affiliated Lianyungang Hospital of Xuzhou Medical University, Lianyungang, China; ^2^Department of Neurology, Jinzhou Medical University, Jinzhou, China; ^3^Department of Clinical Laboratory, Lianyungang Oriental Hospital, Lianyungang, Jiangsu, China; ^4^Department of Neurology, Affiliated Hospital of Kangda College of Nanjing Medical University, Lianyungang, China

**Keywords:** nitrous oxide, subacute combined degeneration of the spinal cord, cognitive decline, vitamin B_12_, methylprednisolone

## Abstract

**Background:**

Nitrous oxide (N_2_O) is an increasingly popular recreational drug. N_2_O irreversibly disturbs the metabolism of vitamin B_12_, resulting in a functional deficiency. Vitamin B_12_ is vital for myelin synthesis and its deficiency primarily produces neurological complications. Inhaling N_2_O is more common and neurological complications are more evident than before.

**Case presentation:**

We report a young man who developed progressive limb numbness and unsteady walking after N_2_O abuse. The dominant diagnosis was subacute combined degeneration of the spinal cord (SCD). The patient was admitted to the hospital and given adenosylcobalamin treatment, but his symptoms progressed significantly from before and he developed acute cognitive impairment. After methylprednisolone combined with vitamin B_12_ treatment, symptoms significantly improved.

**Conclusion:**

Clinicians need to understand the presentation and treatment of SCD caused by N_2_O abuse. When symptoms progress despite conventional vitamin B_12_ therapy, the combination of methylprednisolone and vitamin B_12_ may be considered.

## Introduction

Nitrous oxide (N_2_O), also called “laughing gas,” is a colorless gas with a sweet taste and good stability. Its role as an inhaled anesthetic is primarily in dental and labor analgesia. Because laughing gas inhalation can produce euphoria, it is widely prevalent among young people who are blindly seeking excitement. Long-term abuse can cause severe neurological complications. In recent years, Smoking laughing gas has become increasingly popular, and as a result, neurological complications will be more evident than before. This case reports an adolescent patient with central and peripheral nervous system involvement and acute cognitive decline caused by long-term inhalation of N_2_O. The patient's condition changes and treatment options are described in detail to improve clinicians' awareness of recreational N_2_O abuse.

## Case report

An 18-year-old man was admitted to the emergency center with progressive numbness in the limbs for 10 days. The patient developed numbness in both feet, which gradually progressed proximal end, with numbness in both lower limbs and hands, a sense of girdle in the front chest and abdomen, and a feeling of soreness in the back. After 3 days of admission, the patient's condition progressed significantly compared with the previous. He presented with acute cognitive impairment and weakness in both lower extremities. Without support, he could not walk or stand.

The patient had a history of inhaling N_2_O for 6 months (N_2_O canned, 2 L/can, 2–8 L can be used at a time), 3–4 times/week. The last time he consumed about 10 L was significantly increased compared to the previous time.

### Neurological examination

Clear consciousness, slow language, decreased calculation and orientation, recent memory decline (cannot recall what you ate for breakfast), blunt response and no abnormality were found in the examination of twelve pairs of cranial nerves. The muscle strength of both upper limbs was grade 4, and the muscle strength of both lower limbs was grade 3, the muscle tension was slightly increased, bilateral superficial paresthesias, the sense of position and vibration of both feet were weakened, needle-punching in both feet, inaccurate finger-nose test, unstable heel and knee shin, positive Romberg's sign, weakened tendon reflexes on both sides, involuntary stretch-like movements of both upper extremities, skin scratch test positive, no elicitation of bilateral Barthel's sign and no abnormal meningeal irritation sign.

### Laboratory examination

Homocysteine 58.9 μmol/L (normal value 5–15 μmol/L), vitamin B_12_ (>1,144.0 pg/ml; normal value 200–900 pg/ml considered to be related to taking drugs before admission), folic acid 17.39 nmol/L (normal value is 7–45.1 nmol/L), and no abnormality was found in the rest.

### Magnetic resonance imaging (MRI)

**Figure 1:**
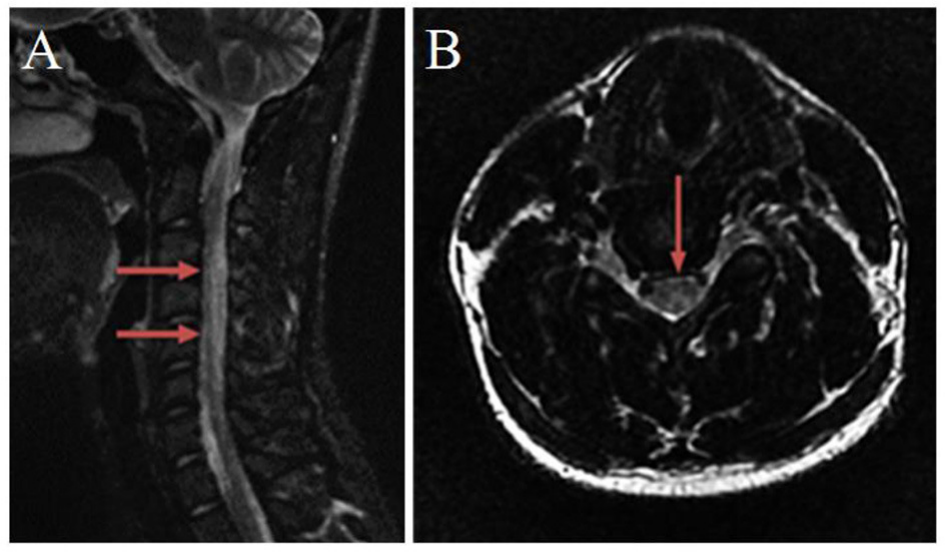
Magnetic resonance imaging of the cervical spinal cord. **(A)** T2-weighted sagittal image, from C2 to C7 spinal cord hyperintensity. **(B)** T2-weighted axial image, an “inverted V-sign” was shown.

MRI of the spinal cord showed the diffuse high signal of the T2W1 sequence (Figure 1A) and the posterior cord of the spinal cord was mainly involved in the axial image, showing an “inverted V sign” (Figure 1B). There were no apparent abnormalities in the thoracic and lumbar spine. There was no obvious abnormality in the head MRI.

### Electroneuromyography (EMG)

Peripheral nerve injury, the lower extremities became more significantly involved than the upper extremities.

The Mini-Mental State Examination Scale (MMSE) scored 18 points (5 points for orientation, 1 point for calculation, 5 points for memory, and 7 points for language ability).

Treatment was given with intramuscular Adenosylcobalamin (1.5 mg/day), and symptoms were further aggravation. The patient was given intramuscular injections of Adenosylcobalamin combined with Methylprednisolone intravenous infusion (500 mg/day for a 5-day course). Adenosylcobalamin (1.5 mg/day) was administered intramuscularly for 10 days. After 7 days of treatment, the patient's chest discomfort in the front, back soreness and numbness in limbs improved, and the orientation, calculation, and mental were improved. The patient was hospitalized for a total of 10 days. At the time of discharge, the muscle strength of his extremities was better than before. The muscle strength of his lower extremities was Grade 4, and that of his upper extremities was Grade 5. Oral medication and rehabilitation after discharge. After 1 month, the patient could walk independently. The Mini-Mental State Examination (MMSE) scored 27 points (8 points for orientation, 4 points for calculation, 6 points for memory, and 9 points for language ability). After 3 months of follow-up, the patient's limb numbness was significantly improved, and his daily life was not affected.

## Discussion

Neurological complications from N_2_O inhalation have been rare before. In recent years, more N_2_O abuse complications have been reported. Inhaling N_2_O can create a relaxing feeling and is relatively easy to obtain. There are rich ways to buy it in the market and the price is low. More young people relax and indulge by inhaling large amounts of laughing gas. However, they are not aware of the possible side effects of inhaling. The extent of N_2_O abuse is often difficult to quantify accurately, most people hide their history of N_2_O use, so N_2_O abuse is often severely underestimated.

To date, the poisoning mechanism of N_2_O has not been fully elucidated. Methylcobalamin in vitamin B_12_ converts homocysteine to methionine, and S-adenosylmethionine, a metabolite of methionine, is irreplaceable for the formation and maintenance of myelin sheaths. Vitamin B_12_ deficiency leads to impaired myelin synthesis and methylation of myelin proteins (1), causing neural demyelination changes. N_2_O interferes with the metabolic pathway of vitamin B_12_ by irreversibly oxidizing the cobalt element of vitamin B_12_, leading to a decrease in vitamin B_12_ (2) and ultimately impaired myelin synthesis and neurological complications. N_2_O interferes with the metabolism of intracellular vitamin B_12_, while serology tests the level of extracellular vitamin B_12_. In the early stages of the disease, in people with a normal diet or with self-supplementation of vitamin B_12_, serum levels of Vit B12 may be normal, but the increase of homocysteine can indirectly reflect the lack of *in vivo* vitamin B_12_ functionality (3).

The patient has been inhaling laughing gas for 6 months, and the body does not have enough stored vitamin B_12_. The patient once took vitamin B_12_ drugs orally, which increased the level of vitamin B_12_ in the blood. Therefore, the serum vitamin B_12_ test was beyond the normal range. And the last time, he inhaled a huge amount of N_2_O, which caused N_2_O toxicity.

The patient had decreased sense of position and vibration of the feet, involving the lamella and wedge tracts, and developed sensory ataxia. The patient's walking instability, inaccurate finger-nose test, and positive Romberg's sign suggest that the lesion involves the spinocerebellar tract. The diffusivity of N_2_O is good, after inhalation, the partial pressure of oxygen in the alveoli can be reduced quickly, resulting in the reduction of oxygen delivered to the brain, resulting in brain hypoxia (4). The patient's muscle tone was slightly increased on admission, accompanied by involuntary stretch-like movements of both upper limbs, which may be related to the extrapyramidal symptoms of basal ganglia hypoxia after a large amount of N_2_O inhalation. The previous literature reported generalized dystonia and involuntary movements for patients with N_2_O abuse, which disappeared after vitamin B_12_ supplementation, suggesting that dyskinesia may be related to neurotoxicity (5).

The patient, in this case, has decreased calculation, spatial and temporal orientation and decreased memory, which is considered to be related to the cognitive dysfunction caused by N_2_O inhalation. Dreyfus et al. (6) reported 2 cases of anesthesiologists with prolonged exposure to N_2_O who experienced cognitive declines such as unresponsiveness, memory loss, and distraction. After stopping work and receiving professional treatment, the appeal symptoms were relieved. Shen et al. (7) described a patient with acute cognitive decline due to long-term inhalation of N_2_O who recovered well after adequate vitamin B_12_ supplementation.

According to the patient's N_2_O abuse history, clinical manifestations and signs, elevated homocysteine, MRI showed an inverted “V” sign, EMG showed limb nerve damage, N_2_O abuse-induced SCD and acute cognitive impairment were diagnosed.

There is no specific treatment protocol for neurotoxicity due to N_2_O abuse and it is mainly based on previous reports in the literature. In our case, the patient's clinical symptoms significantly progressed despite vitamin B_12_ supplementation. Hormones can alleviate spinal cord edema and also have neuroprotective effects, so we used hormones in combination with vitamin B_12_ to rapidly reverse the neurological damage caused by N_2_O abuse. Previous studies have proposed that methylprednisolone decreases desynovial myelination and axonal damage (8). It also promotes the survival of neurons and supports myelin regeneration (9). Early rehabilitation is also essential for the recovery of nerve function and can vastly reduce the extent of nerve damage (10). When there is abnormal mental behavior, we should also pay attention to effective psychological counseling, give patients active psychological support treatment, encourage patients to stay away from N_2_O, and develop healthy work and living habits.

## Conclusion

In short, the clinical manifestations caused by long-term inhalation of N_2_O are different. Clinicians should have sufficient knowledge of the clinical manifestations and treatment of N_2_O toxicity. Clinically, when patients complain of neurological complications such as numbness of limbs, unstable walking, and weakness of limbs, especially in adolescents, clinicians should inquire whether they have a history of inhaling N_2_O. The young patient with acute cognitive impairment should be associated with the possibility of N_2_O poisoning. When symptoms progress despite treatment with vitamin B_12_ supplementation, a combination of methylprednisolone and vitamin B_12_ may be considered.

## Data availability statement

The original contributions presented in the study are included in the article/supplementary material, further inquiries can be directed to the corresponding authors.

## Author contributions

HW: data analysis, interpretation, and drafting of the manuscript. HH, LX, and NJ: critical revision of the manuscript. XZ: study concept and design and critical revision of the manuscript. KX: study concept and design and study supervision. All authors read and approved the final manuscript.
